# Using a thermistor flowmeter with attached video camera for monitoring sponge excurrent speed and oscular behaviour

**DOI:** 10.7717/peerj.2761

**Published:** 2016-12-13

**Authors:** Brian W. Strehlow, Damien Jorgensen, Nicole S. Webster, Mari-Carmen Pineda, Alan Duckworth

**Affiliations:** 1 Centre for Microscopy, Characterisation and Analysis, School of Plant Biology, and Oceans Institute, University of Western Australia, Crawley, WA, Australia; 2 Western Australian Marine Science Institution, Crawley, WA, Australia; 3 Australian Institute of Marine Science, Townsville, QLD, Australia

**Keywords:** Flowmeter, Sponge, Thermistor, Pumping, Behaviour, Contraction

## Abstract

A digital, four-channel thermistor flowmeter integrated with time-lapse cameras was developed as an experimental tool for measuring pumping rates in marine sponges, particularly those with small excurrent openings (oscula). Combining flowmeters with time-lapse imagery yielded valuable insights into the contractile behaviour of oscula in *Cliona orientalis*. Osculum cross-sectional area (OSA) was positively correlated to measured excurrent speeds (ES), indicating that sponge pumping and osculum contraction are coordinated behaviours. Both OSA and ES were positively correlated to pumping rate (*Q*). Diel trends in pumping activity and osculum contraction were also observed, with sponges increasing their pumping activity to peak at midday and decreasing pumping and contracting oscula at night. Short-term elevation of the suspended sediment concentration (SSC) within the seawater initially decreased pumping rates by up to 90%, ultimately resulting in closure of the oscula and cessation of pumping.

## Introduction

Marine sponges (Porifera), and their associated microbial symbionts, perform many ecologically important functions including: creating and bioeroding substrate, coupling bentho-pelagic biogeochemical fluxes (e.g., carbon, nitrogen, silicon, and phosphorus cycling), forming intricate associations with other organisms (Bell, 2008; Maldonado, Ribes & Van Duyl, 2012), and converting dissolved organic matter to particulate organic matter for use in oligotrophic food webs (De Goeij et al., 2013; Maldonado, 2016). Furthermore, marine sponges are present in a wide variety of habitats from the polar regions to the tropics and from the shallows to the deep sea (Van Soest et al., 2016), and they are often the most abundant, macrobenthic taxa present (Flach et al., 1998; Diaz & Rützler, 2001; Heyward et al., 2010).

Sponges are generally considered ‘simple’ animals, yet they have surprisingly complex physiologies. Sponges actively pump water through their tissues using specialised chambers of flagellated cells called choanocytes. Water is pumped through small incurrent pores (ostia) to a system of channels leading to chambers full of choanocytes before flowing out via larger excurrent openings (oscula) (Bergquist, 1978). They depend on this water circulation for most of their essential physiological processes, i.e., food capture, waste elimination, gas exchange and reproduction (Bergquist, 1978). Sponges filter large volumes of water, with retention efficiencies between 75 and 99% for particles and biota of various sizes (Reiswig, 1971; Reiswig, 1975; Pile et al., 1997). Sponge populations can filter the equivalent volume of the overlaying water column in <1 to 56 days, depending on density, water depth and community composition (Reiswig, 1974; Pile et al., 1997; Savarese et al., 1997).

Despite the global abundance and ecological importance of sponges, sponge physiology is understudied, comprising <1% of published literature on sponges (Becerro, 2008). Currently, pumping rates (*Q*) have been measured for <0.01% (Weisz, Lindquist & Martens, 2008; Massaro et al., 2012; McMurray, Pawlik & Finelli, 2014) of the approximately 8,700 described species (Hooper & Van Soest, 2002; Van Soest et al., 2016). Sponge pumping is highly variable depending on the species, being continuous with occasional, temporary cessation for species with thick tissue layers (Reiswig, 1971; Reiswig, 1974; McMurray, Pawlik & Finelli, 2014) or periodic in species that are more behaviourally active (Reiswig, 1971; Reiswig, 1974). Generally, sponges with high microbial abundances (HMA) have lower pumping rates than low microbial abundance (LMA) species (Weisz, Lindquist & Martens, 2008). Sponge behaviour is also highly variable depending on the species. Some species can actively open and close their ostia and oscula (Reiswig, 1974; Harrison, 1972; Weissenfels, 1976; Ilan & Abelson, 1995; Leys & Meech, 2006; Elliott & Leys, 2007); expand and contract aquiferous systems and pinacoderm (outer layer) (Nickel, 2004; Ellwanger & Nickel, 2006; Nickel et al., 2011); and even execute coordinated behaviour patterns (Leys & Meech, 2006; Ludeman et al., 2014).

Contractile behaviours have only been directly linked to sponge pumping rates in two species: *Tectitethya crypta* and *Verongia reiswigi* (*Tethya crypta* and *V. gigantea* in Reiswig, 1971; Reiswig, 1974). *T. crypta* opens its oscula, increasing its osculum cross-sectional area (OSA) during the day and decreasing it at night. The increased OSA corresponds to increased pumping rates during the day, which drop off after sunset as the oscula close. *V. reiswigi,* on the other hand, exhibits steady pumping with periodic cessations. During these cessations, the excurrent pores that channel water into the atrium of the osculum close (Reiswig, 1971).

An enhanced understanding of how sponge physiology and ecophysiology respond to anthropogenic stressors will be particularly valuable in the current period of rapid environmental change. Previous studies have shown that pumping rates in sponges significantly decrease or arrest when exposed to elevated SSCs (Reiswig, 1971; Gerrodette & Flechsig, 1979; Leys, Mackie & Meech, 1999; Tompkins-MacDonald & Leys, 2008). However, it remains to be seen if this response is ubiquitous across the Porifera, as it has only been reported in a few species. During dredging operations, where sediments are removed from the seabed to either maintain or create ports, SSCs can range from 100–300 mg L^−1^ for periods of several hours and from 10–30 mg L^−1^ over several days in areas within 500 m of dredging activities (Jones et al., 2015). The physiological and ecological impacts of sediments at these levels on sponges, even for those species well adapted to turbid environments, remain largely unknown (Bell et al., 2015; Schönberg, 2015; Schönberg, 2016).

In order to elucidate the relationship between sponge oscula opening/closing behaviour and pumping rate, we examined the common, Indo-pacific bioeroding sponge *Cliona orientalis* (Thiele, 1900). This species contains phototrophic, intracellular symbionts (*Symbiodinium* sp.) (Schönberg & Loh, 2005), and it can open and close its oscula (Schönberg, 2000), consistent with other Clionaids (e.g., Grant, 1826; Friday, Poppell & Hill, 2013, B Strehlow, pers. obs., 2012 *Cliona viarians*). *C. orientalis* exhibits increased bioerosion during the day as well as a diurnal change in symbiont distribution (Fang et al., 2016b), which indicate that, like *T. crypta*, it could have a diurnal, physiological rhythm. However, the pumping and behavioural patterns of *C. orientalis* have not yet been described over time.

The central aim of this study was to characterise the pumping and behaviour of *C. orientalis*, which was achieved by developing a multi-channel digital thermistor flowmeter that incorporates time-lapse cameras to observe oscula behaviour and excurrent speeds (ES) concurrently. These values were used to calculate pumping rates under ambient environmental conditions. The pumping rate was hypothesised to be periodic and not continuous, hence the relationship between ES and OSA during contractions was also examined. It was further hypothesized that a positive correlation existed between ES and OSA and that *C. orientalis* pumping would follow a diel pattern, increasing during the day and decreasing at night. In addition, the flowmeter and camera apparatus was used to determine the pumping and contractile responses of *C. orientalis* to elevated SSCs from both single and multi-pulse events. It was predicted that *C. orientalis* pumping rates would decrease in response to elevated SSCs comparable to those of a dredging plume.

## Materials and Methods

### Thermistor flowmeter construction and calibration

A four-channel thermistor flowmeter with integrated video camera was designed, constructed and validated in order to simultaneously measure sponge excurrent speed and observe sponge behaviour. The thermistor flowmeter was designed and built at the Australian Institute of Marine Science (AIMS) in Townsville with all testing done at the AIMS National Sea Simulator (SeaSim). The flowmeter incorporated four independent thermistor probes and a thermometer to determine ambient temperature. Each glass thermistor probe (120 series, 1,000 Ω at 25 °C, Honeywell, USA) was set to hold at 10 °C above ambient temperature. In high flow conditions, the probes drew more power to maintain this temperature difference hence power could be directly correlated to flow rate. This design is similar to thermistor flow meters used by Reiswig (1971) and Labarbera & Vogel (1976); however, in the current model, measurements were recorded digitally and four probes could measure flow simultaneously.

For calibration, each probe was attached to a carriage running along a rail on top of the tank and driven by a stepper motor through a belt and pulley system. The probes were run across the track at 8 different speeds from 5 to 200 mm s^−1^. Three calibration runs were performed for each speed, and the power used to maintain thermistor temperature was averaged across the three trials. To confirm precision at different temperatures, the same calibration runs were performed between 24–29 °C (at 1 °C intervals). Curves were fitted to a polynomial regression model for power compensation developed by Moore (2003) using R64 (R Core Team, 2013). R-squared values for polynomial regression models for all four probes calibrated at four temperatures were greater than 0.99. A successive approximation method based on the calibration curve was used to back-calculate flow rate from power output. Custom designed software facilitated modification of the calibration equations according to temperature. The reliable range of the instrument was defined as 5–200 mm s^−1^ with an accuracy of ±5 mm s^−1^.

As a consequence of having the probes heated to 10 °C above the ambient temperature, no clear trend could be determined between ambient temperature and power, with curves from different temperatures intersecting at two points. Under identical flow conditions, differences in measured flow of 5 mm s^−1^ could be recorded if temperatures changed by >1 °C. Experiments were therefore conducted at a controlled temperature for which a specific calibration curve was generated. As the logging software records changes in temperature to ±0.01 °C, experiments can be monitored effectively, quality controlled and were aborted if temperatures changed by ≥0.5 °C.

### Sample collection and experimental setup

**Figure 1:**
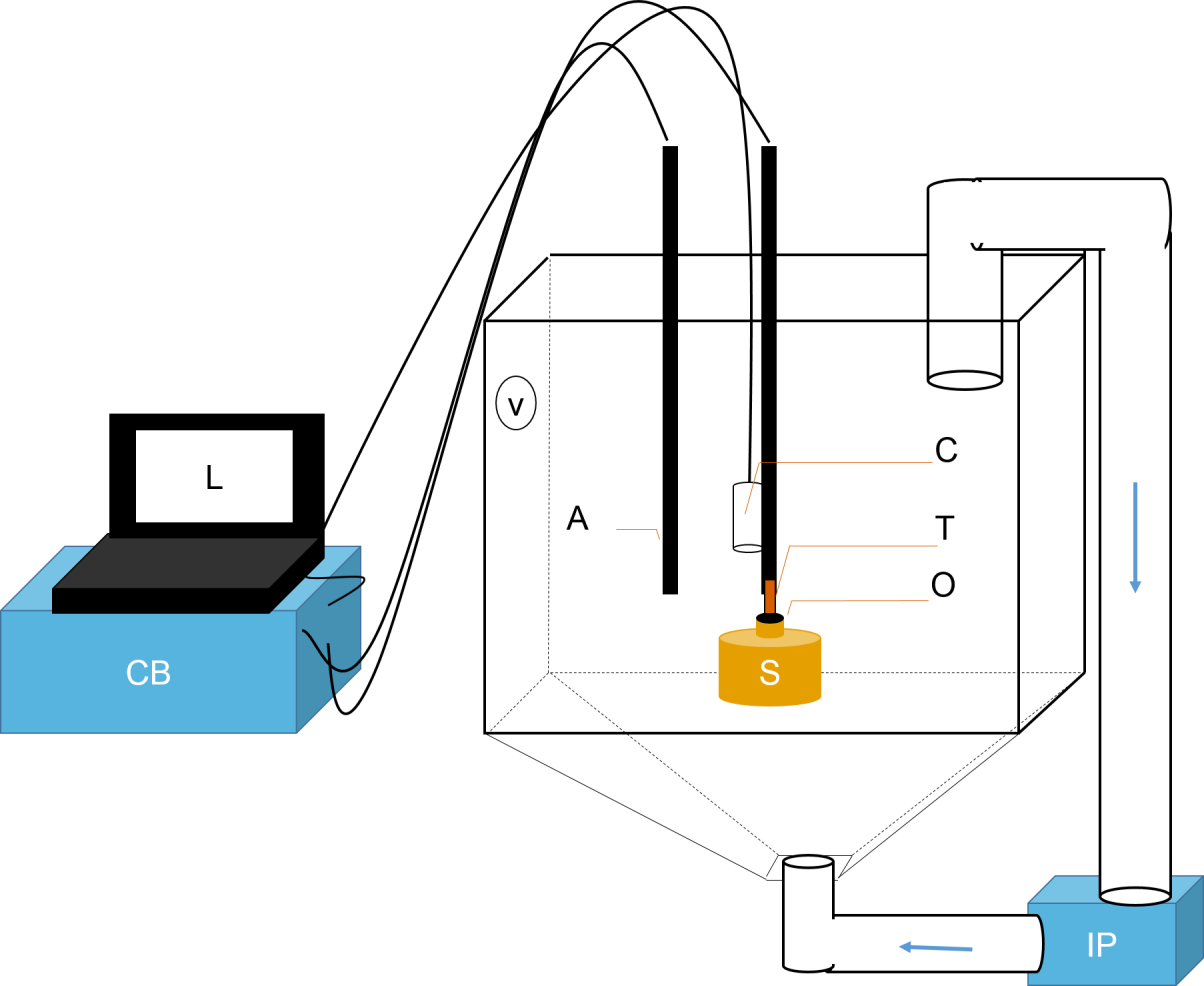
Schematic representation of flowmeter and experimental tank. A laptop computer (L) controlled the time-lapse USB camera (C). The ambient temperature probe (A) and the thermistor flowmeter (T) were controlled by the control box (CB). Data was sent from CB to L for processing. T was positioned directly over sponge (S) osculum (O). Sediments were kept in suspension using two pumps: a Vortech MP10 (V) and an Iwaki MD (IP). The latter recirculated water and sediments from below (flow direction is shown with blue arrows).

Small sponge explants (50 mm in diameter and containing several oscula) air drilled from large colonies of *C. orientalis* inhabiting (bioeroding) the skeleton of dead *Porites* sp. coral. Explants were collected at a depth of 3 m from reefs around Pelorus Island on the inshore Great Barrier Reef (GBR) (S18°32.903′ E146°29.172′, GBR Marine Park Authority Permit:G13/35758.1) and were acclimated to aquarium conditions for two weeks before experimentation. Aquaria consisted of 100 L tanks (Fig. 1) held at 27 °C. Seawater (5 µm filtered) flowed into tanks at 600 mL min^−1^ (∼9 turnovers per day) ensuring abundant food and nutrients for the animals. Water in each tank was recirculated using an Iwaki MD magnetic drive centrifugal pump (Iwaki Pumps, Sydney, Australia) that collected water from the surface and forced it up through the centre point of the inverted pyramid at the tank’s base (Fig. 1A, IP) in conjunction with a VorTech MP10 pump (EcoTech Marine, Allentown, PA, USA) mounted inside the tank (Fig. 1, V). Hydra 52 LED lights (AquaIllumination, Ames, IA, USA) ramped up to 200 µmol photons m^−2^ s^−1^ from 5:30 to 11:30, held at the maximum for one hour and then decreased to 0 from 12:30 to 17:30. This natural daily pattern of light provided sufficient intensity for the phototrophic *C. orientalis*.

### Observations of oscula behaviour, ES, OSA and Q

Waterproof USB endoscopes (7 mm USB waterproof endoscope; Shenzhen Dowdon Tech Co, Shenzen, China) aided in accurate positioning of the probes over the oscula (item C in Fig. 1). 3D flow patterns were measured for a single osculum. The probe was positioned in three dimensions around the osculum, with flow rates measured for 1 min per location. ESs across the osculum plane were integrated gravimetrically based on the area measured to determine the mean ES over the entire OSA (Reiswig, 1974). The ratio of the mean ES to the centreline ES (ES_centre_), i.e., the ES recorded at the centre of the OSA, was 0.90, and this value was used as the profile correction factor (see Eq. (2)), which accounted for any decrease in speed at the edge of the osculum (Reiswig, 1974; McMurray, Pawlik & Finelli, 2014). Based on 3D measures of ES, the excurrent stream was considered to be laminar with a plug-like flow profile.

The OSA and centreline ES (ES_centre_) were measured for all oscula (*n* = 10) on two different sponge explants (E1 and E2) for approximately 40 min per oscula. Reported measurements of ES should be considered as ES_centre_ unless otherwise specified. The OSA was based on the diameter of the osculum as measured by ImageJ (Schneider, Rasband & Eliceiri, 2012), with area calculated assuming a circle. Reduced major axis (RMA) regressions were performed using the lmodel2 package in R64 (R Core Team, 2013) to correlate OSA to ES_centre_ (*sensu* McMurray, Pawlik & Finelli, 2014). Additionally one-way Analyses of Variance (ANOVAs) were run to determine if the ratio of ES: OSA was different between oscula on the same explant.

At each time point, the instantaneous pumping rate (*Q*), i.e., “point rate” (Reiswig, 1974), was calculated as follows:

(1) \begin{eqnarray*}Q=\mathrm{OSA}\times {\mathrm{ES}}_{\mathrm{centre}}\times \text{profile correction factor (Adapted from Reiswig, 1974)}.\end{eqnarray*}

RMA regressions were also performed to relate OSA to *Q* and ES to *Q*. This equation was used to approximate *Q* based on ES in future experiments:

(2) \begin{eqnarray*}Q=5.42\times 1{0}^{-3}\mathrm{(ES)}^{1.60}\end{eqnarray*}

Mean *Q* values for each osculum were summed to calculated specific pumping rate based on explant volume. Explant volume was approximated by measuring the surface area on the top of the core and the depth that the sponge eroded into the substrate and calculating the volume of the cylinder.

### Effects of elevated SSC

If probes were exposed to elevated SSCs, a layer of sediment deposited on them over time. This layer significantly decreased the probe readings. To counteract this effect, probes were cleaned twice a day using small burst of water from a submerged transfer pipette. Two experiments were run exposing *C. orientalis* to elevated SSCs: (i) a single, short term exposure and (ii) a long term exposure with three pulses of sediment. Sediments were prepared from biogenic calcium carbonate sediment collected from Davies Reef (a clear-water, mid-shelf reef in the central GBR). Sediment was dried and ground using a rod mill grinder until the mean grain size was ∼29 µm with 80% of the sediment ranging in size between 3 and 64 µm, as measured using laser diffraction techniques (Mastersizer 2000; Malvern Instruments Ltd, Malvern, UK). Suspended sediments were added to the experiments gradually (over approximately five min) to reach a level of 100 mg L^−1^. This level was chosen to reflect SSC within 500 m of dredging activities, where SSCs exceeded 100 mg L^−1^ for periods of several hours (Jones et al., 2015).

In the short term SSC exposure, four explants (E3–6) were tested individually on separate days. Each of the four trials was independent, and the tanks were cleaned between trials. For consistency, each trial was conducted at the same time of day. To obtain ‘normal’ pumping rates (i.e., unaffected by sediment), excurrent speeds from each sponge were measured for 30 min. Each sponge explant was subsequently exposed to a single pulse of 100 mg L^−1^ sediment and monitored for 3.5 h. Sediments settled quickly, with SSC decreasing to ∼50 mg L^−1^ in the tanks after 1.5 h and to ∼40 mg L^−1^ after 3 h. Temperature was held constant at 28 °C for explants 3 and 4, and 27 °C for explants 5 and 6. *Q* was calculated using Eq. (2).

In the long term SSC exposure experiment, ES and OSA were recorded for an additional four explants (E7–10) for 2.5 d before and after three separate sediment pulses of 100 mg L^−1^, each applied over a 4 h period. Again, *Q* was approximated using Eq. (2). The diurnal pattern of *C. orientalis* pumping was also assessed. During the first 24 h of observation, diurnal pumping patterns were assessed based on 20 min moving averages of calculated pumping rates.

Since pumping rates oscillated regularly throughout the day, percentage change was recorded based on the local maxima (Reiswig, 1974; Reiswig, 1975). SSCs were monitored using a nephelometer (TPS, Australia), and nephelometric turbidity units (NTUs) converted to SSCs (as mg L^−1^) by applying a sediment-specific conversion factor. Sedimentation rates were measured using SedPods (Field, Chezar & Storlazzi, 2013), which were capped after 24 h in the tank with deposited sediment filtered through pre-weighed 0.4 µm 47 mm diameter polycarbonate filters, incubated at 60 °C for 24 h, and weighed to 0.0001 g to determine sediment mass. Sedimentation rates during this experiment were ∼6 mg cm^−2^ day^−1^. Tank temperature was maintained at 27 ± 0.2 °C during the experiment.

## Results

### Observations of oscular behaviour, OSA, ES and *Q*

**Figure 2:**
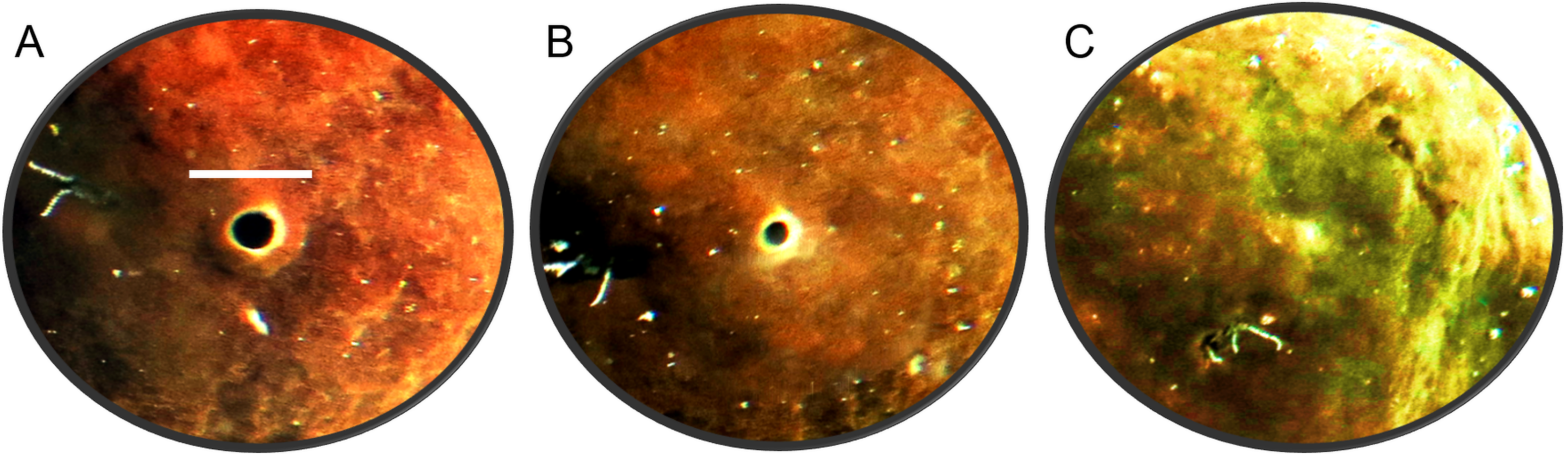
Time-lapse images of *Cliona orientalis* oscular states. (A) Osculum fully open (scale bar: 10 mm). (B) Osculum partially contracted (+10 min) (C) Osculum fully contracted, i.e., closed, following mechanical disturbance.

The oscula of *C. orientalis* contracted and partially closed (Figs. 2A and 2B) on a regular basis. When an osculum closed completely the adjacent area also contracted (Fig. 2C), leaving a small indentation in the tissue. Interestingly oscula on the same explant were observed to undergo partial contractions asynchronously. If a single osculum was disturbed mechanically, it closed and ceased pumping within 2 min. If the whole explant was disturbed or moved within the tank, all oscula on the explant closed within two minutes.

**Figure 3:**
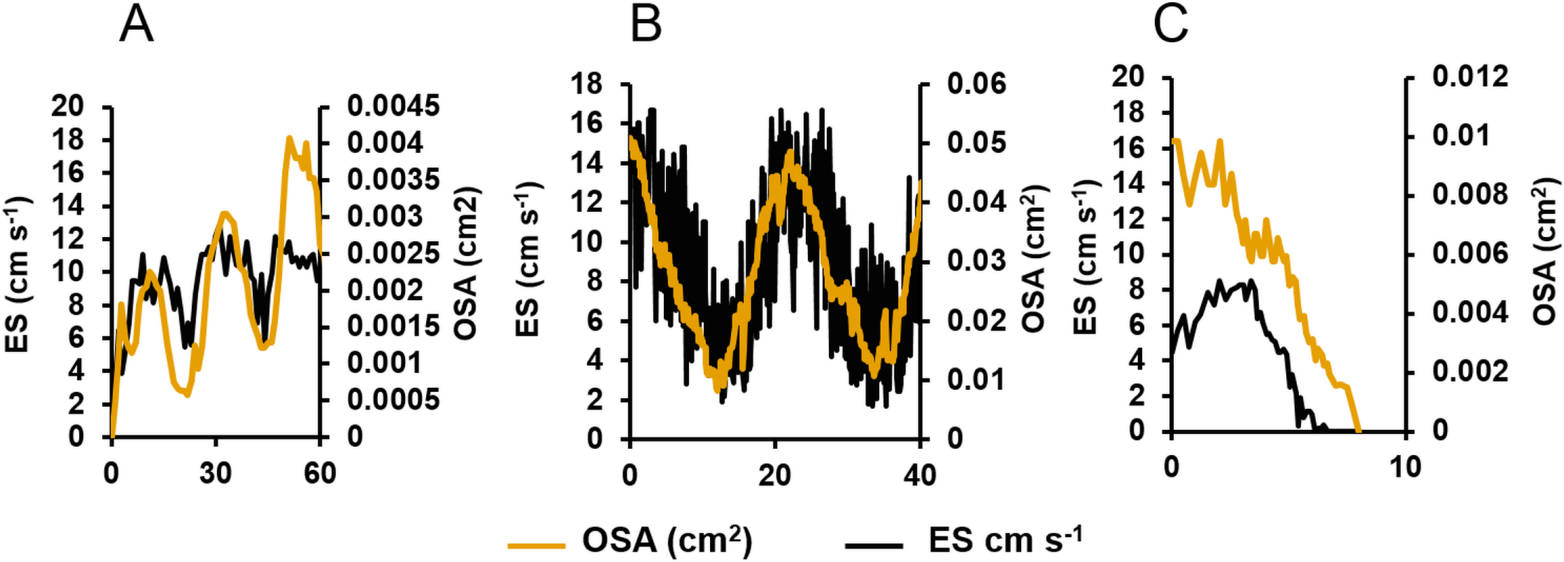
Centreline excurrent speeds (ES) and osculum cross-sectional area (OSA) for three different oscula exhibiting different contractile behaviours. (A) The osculum was actively opening (from a state of complete closure) and initiating pumping. (B) The osculum was open and actively pumping. (C) The osculum was closing and decreasing pumping.

When the oscula opened, the OSA, ES and *Q* increased. Once open, the osculum partially contracted regularly again (decreasing OSA), but it did not fully close (Fig. 3A). These partial contractions coincided with decreased ES. As *Q* is the product of OSA and ES, it follows the same periodic increase and decrease. The same partial contractions occurred when a different osculum was pumping actively (Fig. 3B). Again, *Q* followed a regular period. When a third osculum closed (Fig. 3C), the ES decreased proportionally with a lag of approximately one min.

Summaries of centreline ES, OSA, *Q* and partial contraction period for ten oscula of two separate explants are presented in Table 1. Factoring in all oscular flow rates, explants 1 and 2 had a mean specific volume flow (per litre of sponge tissue) of 0.020 ± 0.008 and 0.007 ± 0.002 L water s^−1^ L sponge^−1^ (mean ± SD). The estimated mean cycle times, i.e., the time required for all oscula to pump a sponge’s volume, were 41 and 151 s, respectively. During active pumping, the osculum partially contracted and then reopened following a regular period of 20.84 ± 2.86 min.

**Table 1:** Summary of centreline excurrent speeds (ES), osculum cross sectional area (OSA), pumping rates (±SD) and contraction periods from ten oscula on two explants of *Cliona orientalis* during different states of contraction: opening, open (with periodic partial contraction), and closing (full contraction).

Explant	Osculum	State	Mean ES (cm s^−1^)	Max ES (cm s^−1^)	Mean OSA (cm^2^)	Max OSA (cm^2^)	Mean pumping rate (mL s^−1^)	Max pumping rate (mL s^−1^)	Osculum partial contraction period (min)
1	1	Open	8.27 ± 3.91	16.66	0.027 ± 0.012	0.052	0.234 ± 0.185	0.720	22.25
	2	Closing	4.83 ± 2.79	8.51	0.006 ± 0.002	0.010	0.031 ± 0.0212	0.076	17.67
	3	Open	7.64 ± 1.49	10.59	0.033 ± 0.006	0.045	0.237 ± 0.081	0.408	19.83
	4	Open	5.74 ± 0.83	7.76	0.140 ± 0.030	0.188	0.723 ± 0.189	1.212	20.86
	5	Open	7.62 ± 2.87	14.45	0.017 ± 0.003	0.021	0.112 ± 0.040	0.188	23.50
2	1	Open	8.33 ± 2.98	12.28	0.016 ± 0.003	0.022	0.129 ± 0.062	0.225	25.91
	2	Opening	9.32 ± 2.36	12.84	0.002 ± 0.001	0.004	0.018 ± 0.011	0.043	21.75
	3	Open	4.69 ± 0.96	6.50	0.010 ± 0.002	0.013	0.041 ± 0.013	0.076	18.67
	4	Open	7.47 ± 1.27	9.38	0.033 ± 0.003	0.037	0.224 ± 0.047	0.291	17.08
	5	Open	4.81 ± 1.61	7.03	0.011 ± 0.001	0.013	0.051 ± 0.020	0.077	N/A

**Figure 4:**
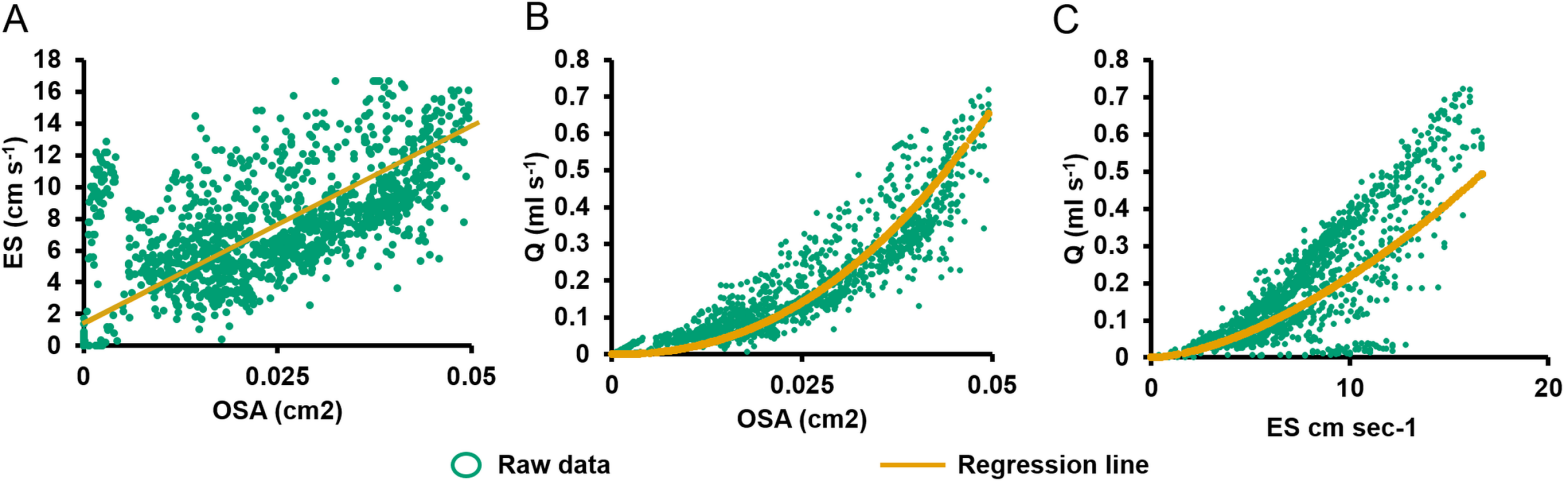
Relationships between pumping variables. (A) Excurrent speed (ES) vs. osculum cross-sectional area (OSA). Regression lines were computed using reduced major axis (RMA) regression. (B) Volume flow (*Q*) vs. OSA. (C) *Q* vs. ES.

During partial contraction, full contraction, and reopening, ES was positively related to OSA (Eq. (3); *R*^2^ = 0.431, *P* < 0.001, *n* = 1,335, Fig. 4A). On closer examination of the ratio of ES to OSA for each oscula, significant differences were demonstrated between values from O2 and all other oscula, O3 and O4, O4 and O1 in E1 (*P* < 0.05). The difference in O2 was due its active closure, whereas all other oscula were undergoing regular partial contraction (Table 1). There are also significant differences between O1 and O3 as well as O3 and O6 in E2. These differences probably account for the ∼50% of variation not explained by the RMA model. *Q* was positively related to OSA (Eq. (4); *R*^2^ = 0.880, *P* < 0.001, *n* = 1,335; Fig. 4B) and ES (Eq. (2), *R*^2^ = 0.946, *P* < 0.001, *n* = 1,335, Fig. 4C). Despite the variation caused by different oscula, both ES and OSA were strongly correlated to *Q*. The regression equations were as follows:

(3) \begin{eqnarray*}ES=250.48(\mathrm{OSA})+1.35\end{eqnarray*}

(4) \begin{eqnarray*}Q=539(\mathrm{OSA})^{2.24}\end{eqnarray*}

### Effects of elevated SSC

**Figure 5:**
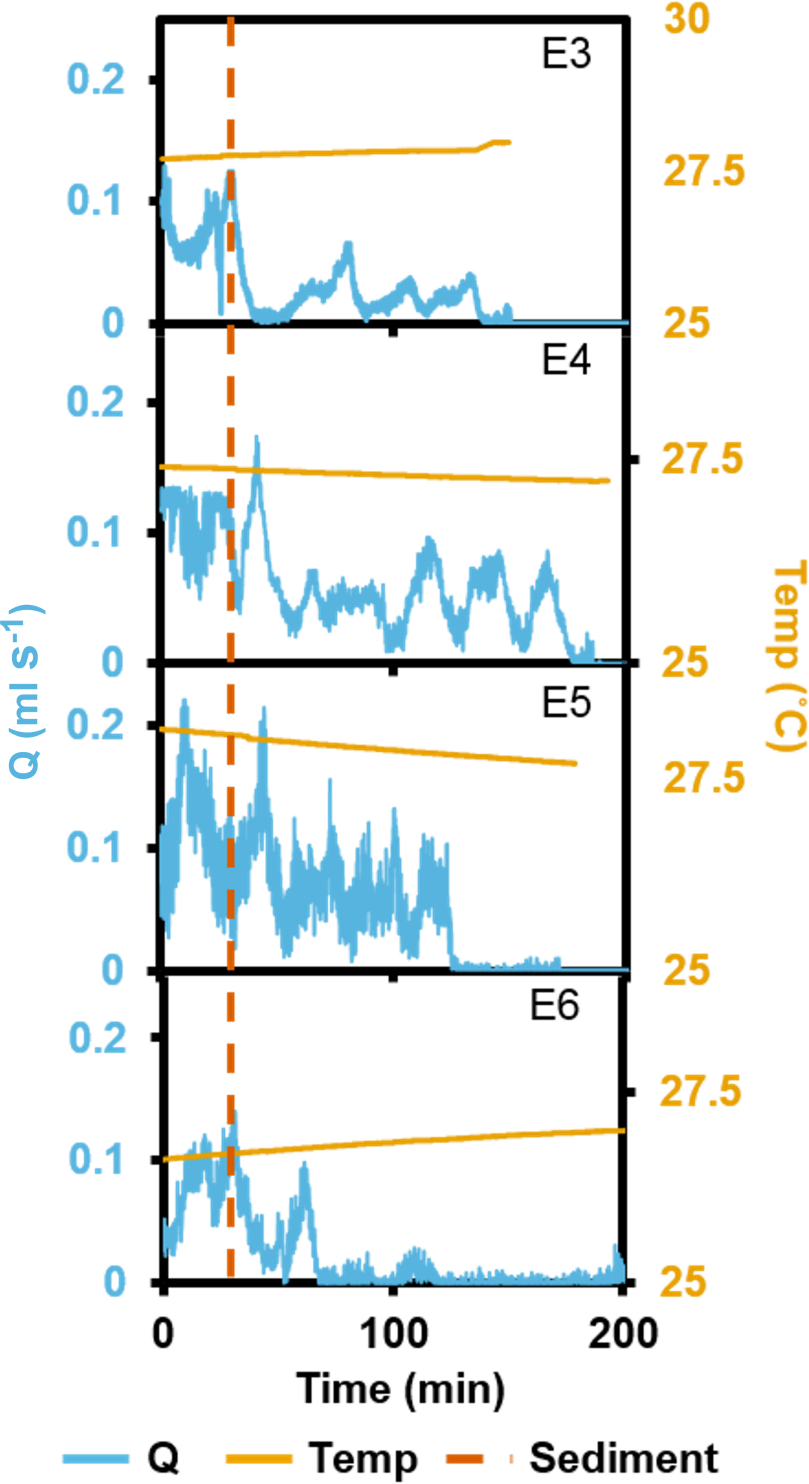
Pumping rates (*Q*) from four oscula of four separate explants (E3–6) exposed to a single dose of high SSC with ambient temperature (temp) recorded. The vertical, dashed line indicates when sediment was introduced.

In the short term SSC exposure experiment, small temperature fluctuations did not have an effect on recorded ESs (Fig. 5). Immediately after exposure to the high SSC, three of the four explants decreased *Q* by 42–90%, and minimum pumping rates were reached after 15 min (Fig. 5). E3 started closing immediately after sediment exposure but partially reopened its osculum and restarted pumping at ∼50% of pre-treatment levels after ∼25 min. E4 exhibited a short burst of increased pumping, and then partially closed its osculum in response to SSC before reducing pumping rates to ∼50% of the pre-treatment level. After 80 mins of reduced pumping, both E3 and E4 closed their oscula and ceased pumping. E5 continued pumping in a “normal,” periodic manner for ∼20 min after exposure, then maximal pumping decreased by ∼50% and osculum closure and pumping cessation occurred after 180 min. E6 closed its osculum and pumping ceased after 30 min. All explants exhibited osculum closure and pumping cessation after treatment. On average, explants kept their oscula open for 99 ± 4 min (mean ± SD) after exposure to sediment. When explants reduced their pumping rates, partial contraction periods of ∼20 min were similar to those explants not exposed to SSC (see Fig. 3).

**Figure 6:**
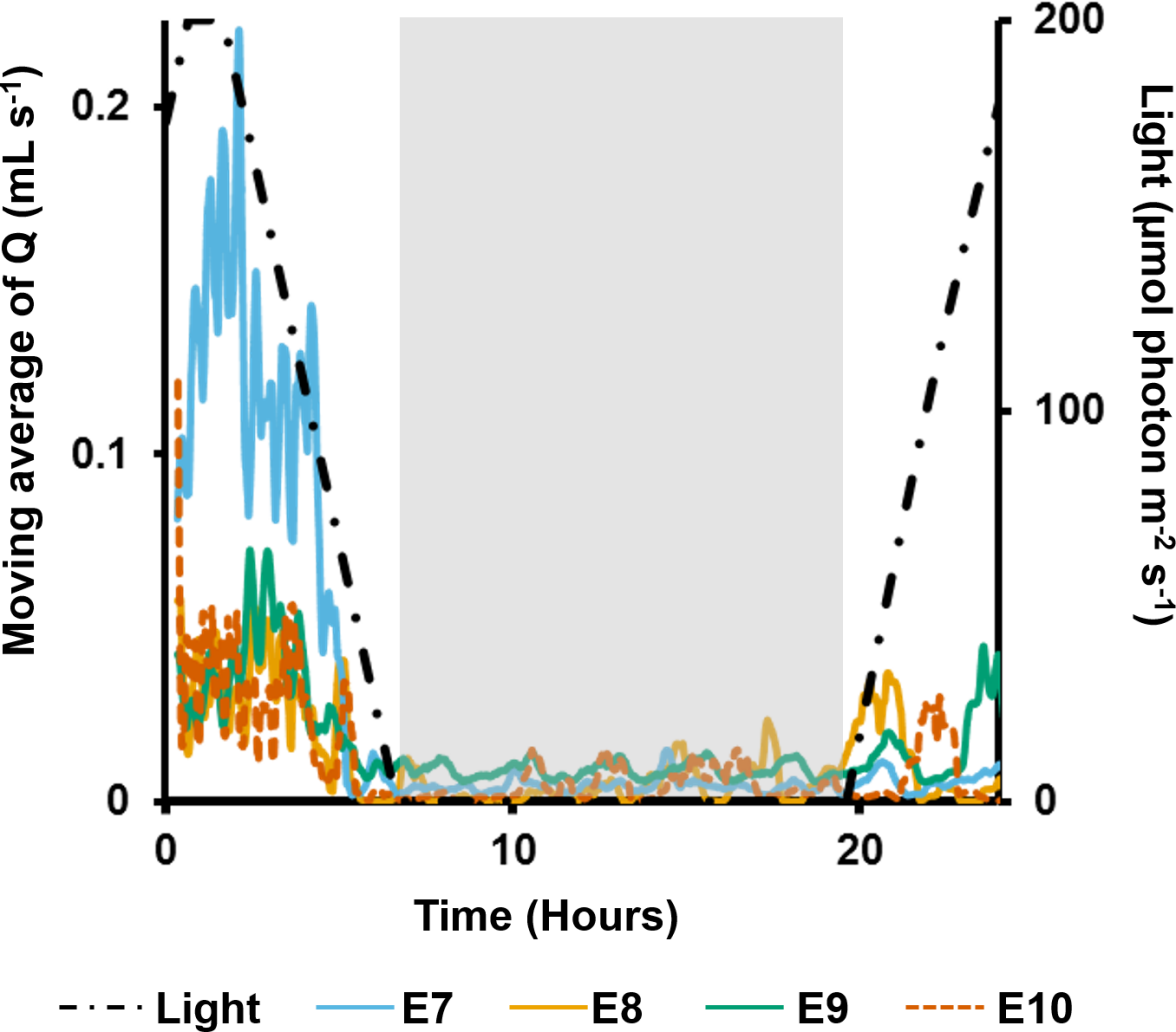
Moving average (period: 20 min) of pumping rates (*Q*) for four explants (E3–6) over 24 h. Light intensity is shown on the secondary *y*-axis, and the grey rectangle represents night.

In the long term sediment dosing experiment, explants 7 to 9 increased pumping rates towards midday, reaching a maximum around the time of highest light intensity (Fig. 6), and then decreased into the night. Although the ∼28 min period of partial contraction of each oscula was consistent throughout the day, excurrent speeds and pumping rates varied during the day. Generally, ES and *Q* increased during the morning to peak approximately 1 h after midday (maximum light intensity), and decreased during the afternoon and were very low during night-time. During the night, pumping activity was markedly depressed and all oscula were either fully or partially contracted. Some pumping occurred at night, but maximal rates were <50% of those during the day.

**Figure 7:**
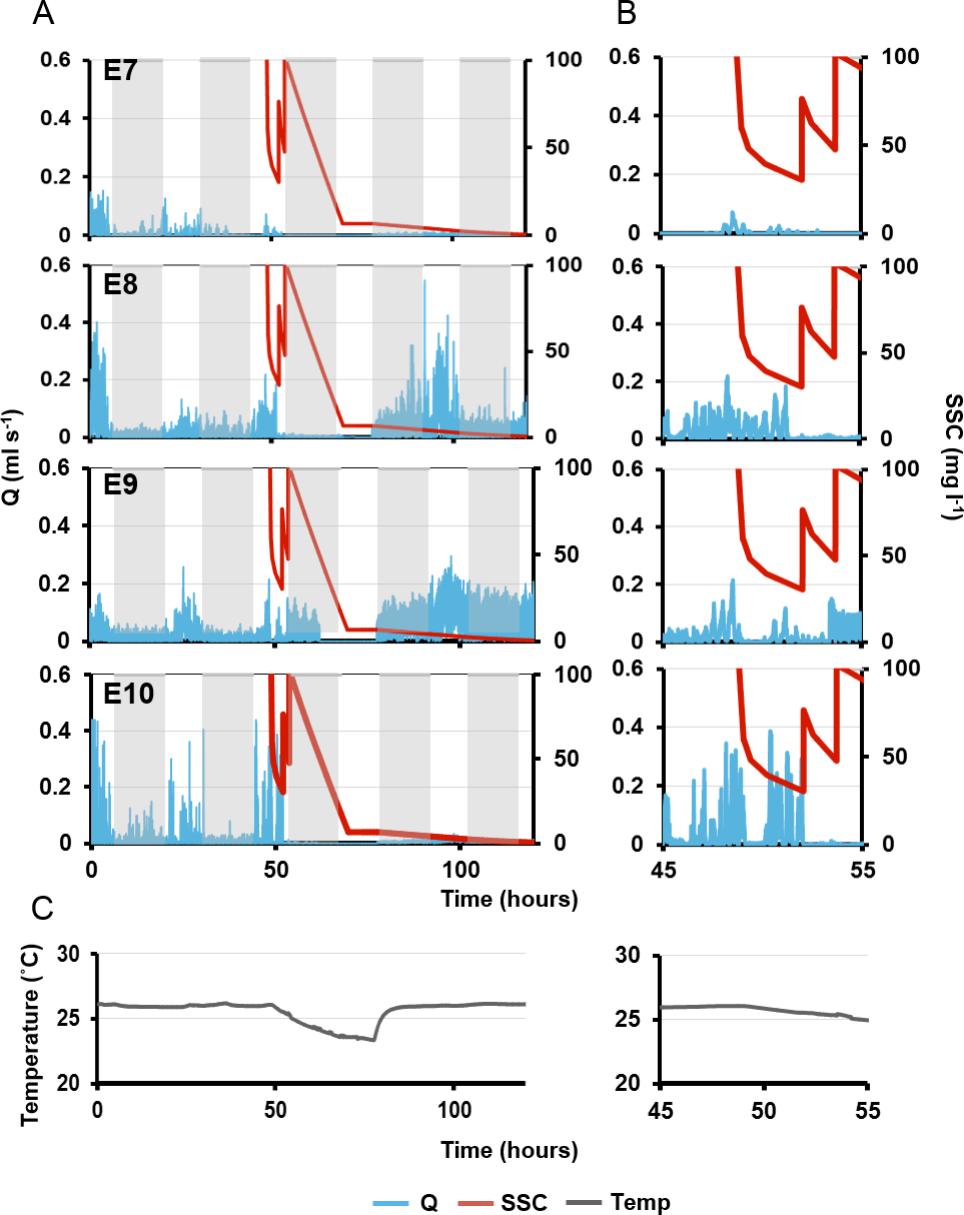
Pumping rates (Q, blue line) in four explants over five days. (A) The grey rectangles represent night. The red line on the third day represents suspended sediment concentrations (SSC) (right-side *y*-axis). Panels E7–10 represent a single osculum of different individual explants measured simultaneously. (B) Close-up of ‘A’ graphs on the third day, when sediment was added. (C) Ambient temperature over time corresponding to times shown in (A) and (B).

On the second day, pumping rates were generally lower and the diel cycle was less obvious, although night-time speeds decreased for all individuals (Fig. 7). The first sediment pulse reduced excurrent speeds for all measured oscula (Fig. 7). Maximal excurrent speed decreased by 41%, 11% and 36% for explants 7–9, respectively (mean: 29 ± 9%). Calculated maximal pumping rates decreased by 57%, 27% and 51%, respectively, with a mean decrease of 41% ± 12% mL s^−1^ (Fig. 7B). In contrast, the maximal excurrent speed of E10 increased by 7%, and pumping rates increased by 12%. All four explants closed their oscula following the first sediment dose after an average of 56 ± 67 min. E9 exhibited a very rapid closure, arresting pumping and closing after 6 min. After the first sediment pulse, all explants except E8, reopened their oscula and restarted pumping 68 ± 26 mins after closure, albeit at lower flow rates than before.

Maximal excurrent speeds after the second sediment dosing decreased by 59  ± 19% from pre-treatment maxima and pumping rates decreased by 71  ± 19%. However, variability was again detected between explants. E7 ceased pumping and closed its osculum after 70 mins. E8 kept its osculum closed. E10 closed its osculum after <1 min, and E9 kept its osculum open even after the second dose (although maximal excurrent speed and pumping rate decreased by 23% and 34%, respectively).

After the third sediment dose, all individuals except E9 closed their oscula and ceased pumping until nightfall. Pumping ceased through the night, in concordance with the diel pumping pattern observed before treatment. E9 kept its oscula open although excurrent speed and pumping rates were reduced by 39% and 55% respectively. Approximately 48 h after the first sediment treatment, E8 and E9 exhibited excurrent speeds and pumping rates similar to pre-treatment levels, and the diel pattern was re-established with maximal excurrent speeds at peak light intensity. A similar pattern was observed in E8, although excurrent speeds did not return to pre-sediment levels. Explant 10 did not restart pumping or reopen its osculum for at least 72 h post-exposure to sediment.

## Discussion

Although sponge pumping behaviour is paramount to sponge biology, physiology and ecology, it has been examined in relatively few sponge species. This study, combining flowmeters with time-lapse imagery yielded useful insights into the contractile behaviours and pumping rates of *Cliona orientalis*. Both OSA and ES strongly correlated to pumping rates and either could be used to accurately calculate *Q* based on Eqs. (1) or (4). Affordable, user-friendly time-lapse cameras could therefore be used in future studies to determine pumping rates in *C. orientalis*. This method, using only cameras, could be easily paired with incubation methods in order to relate respiration, oxygen production, feeding, and/or POM production to pumping rates (e.g., Fang et al., 2013b), provided incubation chambers are transparent. However, the equations provided should only be used for studies of *C. orientalis* pumping rates. Standards would need to be calculated to determine *Q* from OSA in other species with contractile oscula. As *C. orientalis* has multiple oscula per sponge, pumping in all oscula could be observed simultaneously so that specific volume flow rates could be calculated. This calculation was hitherto limited only to sponge species or individuals with one osculum due to logistical limitations.

*C. orientalis* is widely distributed in the Indo-Pacific (Van Soest et al., 2016) and an emerging model organism in the study of the effect of climate change and ocean acidification. As a bioeroder with photosymbionts, *C. orientalis* competes with hard corals for space (Fang et al., 2016a), and it increases its growth and bioerosion rates in response to ocean warming and ocean acidification (Fang et al., 2013a). Given potential decreases in carbonate accretion by corals (Hoegh-Guldberg et al., 2007), *C. orientalis* abundance may increase in the future. Furthermore, sponges in general (Bell et al., 2013) and photosynthetic sponges in particular (Bennett et al., 2016) will likely increase in abundance under projected climate change scenarios. This increase would be concurrent with an increased effect of sponge pumping on ecosystem processes (e.g., element cycling), and could even lead to a positive feedback loop that enhances sponge and algal growth while decreasing coral recovery potential (Pawlik, Burkepile & Thurber, 2016).

It is therefore important to understand the baseline physiology of *C. orientalis.* In this study we report the first specific volume pumping rates for *C. orientalis.* Pumping rates of 0.020 ± 0.008 and 0.007 ± 0.002 L water s^−1^ L sponge^−1^ calculated here were comparable to those of other sponge species (see Weisz, Lindquist & Martens, 2008). *C. orientalis* is classified as a low microbial abundance (LMA) species like *Cliona varians* (Poppell et al., 2013); however, the specific pumping rate of *C. orientalis* was considerably lower than the mean specific pumping rate of other LMA sponges (∼0.27 L water s^−1^ L sponge^−1^) (Weisz, Lindquist & Martens, 2008) and more comparable to the mean specific pumping rate of high microbial abundance (HMA) sponges (∼0.09 L water s^−1^ L sponge^−1^) (Weisz, Lindquist & Martens, 2008; McMurray, Pawlik & Finelli, 2014). *C. orientalis* likely has a lower pumping rate due to its high abundance of eukaryotic photosymbionts (Schönberg & Loh, 2005), compared to other LMA sponges, and consequent reliance on phototrophy over heterotrophic filter feeding (Fang et al., 2013b).

*C. orientalis* pumping rates and oscular behaviour exhibited diel patterns. Pumping rates increased during the morning to peak after noon, decreased during the afternoon and were minimal at night. This coincided with an observed decrease in osculum area at night. The Caribbean sponge *Cliona varians* also contracts its oscula at night (B Strehlow, pers. obs., 2012), as does *T. crypta*, which concurrently decreases its excurrent speed overnight (Reiswig, 1971). This increased activity during the day could explain why *C. orientalis* increases its chemical bioerosion rate during the day compared to night (Fang et al., 2016b). Interestingly, the distribution of endosymbiotic *Symbiodinium* within *C. orientalis* follows a diel rhythm, with algal cells appearing closer to the pinacoderm during the day (Schönberg & Suwa, 2007; Fang et al., 2016b). Changes in pumping activity may be a central part of the diel rhythms of the *C. orientalis* holobiont, as pumping is critical to physiological maintenance of the holobiont. Hence, establishing a direct, causal link between increased pumping rates, increased bioerosion and altered symbiont distribution should be an area of further research.

Osculum area was previously found to be directly correlated with excurrent speed in *Tectitethya crypta* (Reiswig, 1971). Mechanistically, this correlation indicates that either the osculum acts as a passive, elastic valve that closes when no pressure is applied by the pumping choanocytes or that there is communication between choanocytes and contractile pinacocytes in the osculum. According to Ludeman et al. (2014), the osculum itself may now be considered a sensory organ that coordinates behavioural responses, sensing and reacting to decreased pumping by choanocytes. Flow sensing ciliated pinacocytes have been identified in oscula from numerous sponge species and classes (Hammel & Nickel, 2014; Ludeman et al., 2014). Although flow sensing cilia structures have not yet been observed in *C. orientalis*, it is likely that they are present given our results. If so, then the oscula of *C. orientalis* sense low flow from the choanocytes and close in response. This is supported by the lag of approximately one min between pumping cessation and full osculum closure (see Fig. 4C). This is also consistent with the observation that *Xestospongia muta,* which has rigid oscula and excurrent pores, can exhibit full pumping cessation via a coordinated, intrinsically generated decrease in choanocyte activity (McMurray, Pawlik & Finelli, 2014).

Elevated SSCs decreased pumping and eventually caused osculum closure and arrested pumping in *C. orientalis*. The decrease in maximal pumping rates was likely caused by suspended sediments clogging the aquiferous system. However the deposition of sediment may have been a mechanical stimulus that caused the prolonged osculum closure. Partial contraction periods were unchanged, even when pumping rates were reduced. This indicated that periodic, partial contraction is an intrinsic aspect of *C. orientalis* pumping behaviour.

Decreased pumping rates in response to elevated SSCs have been reported in numerous sponge species. In *Aplysina lacunosa*, pumping rates decrease by 41% after exposure to sediment concentrations of ∼100 mg L^−1^ (Gerrodette & Flechsig, 1979) and oscula of *Tectitethya crypta* close rapidly in response to wave action and sand score, with storms causing pumping activity to drop by 27% (Reiswig, 1971). Similarly, *Rhabdocalyptus dawsoni* reduces pumping by 32% within five min after exposure to sediments and can also arrest pumping in response to mild tactile stimulation (Leys, Mackie & Meech, 1999; Tompkins-MacDonald & Leys, 2008). However, not all sponges are equally sensitive to tactile disturbance. The glass sponge *Aphrocallistes vastus* only arrested pumping after a very intense tactile response, i.e., when stabbed with a pipette (Tompkins-MacDonald & Leys, 2008). *A. vastus* also reacted rapidly (within 2 s) to deposited sediment with cessation of pumping lasting for intervals of 30–40 s (Tompkins-MacDonald & Leys, 2008). These intervals are comparatively shorter than what was observed in *C. orientalis,* and this may be due to the ability of glass sponges to rapidly propagate action potentials across syncytial tissue (Leys, Mackie & Meech, 1999).

*C. orientalis* has multiple oscula per individual that contract asynchronously under normal conditions. The level of connection and coordination among oscula on a single sponge and, more importantly, the aquiferous system related to each osculum, remains to be determined for this species. However, sponges with encrusting morphologies and contractile oscula likely have connected (confluent) aquiferous systems (Reiswig, 1975). If disturbed locally, an osculum could contract and flow from associated choanocyte chambers would be channelled to adjacent oscula. However, elevated SSC and sedimentation caused all oscula on *C. orientalis* to contract and close. Although possibly a good short term acclimation strategy to localised disturbance, these reductions in pumping rates and prolonged osculum closures are implicitly detrimental to sponges in the long term. If sediment stressors were applied chronically, decreased pumping could lead to hypoxia, starvation and the inability to effectively eliminate waste. However, the long term effects of dredging-related stressors still need to be determined in future work.

## Conclusion

Understanding the baseline physiology of sponges is increasingly important due to the demonstrated ecological importance of sponges in oligotrophic food webs and the changes in coral reef community composition forecasted with increasing anthropogenic stress, e.g., dredging. Here we have elucidated diurnal previously unknown patterns in pumping and contractile behaviour in *Cliona orientalis*. The correlation of OSA to *Q* will provide a valuable metric for future studies of *C. orientalis* physiology. Finally, acute exposures to elevated SSCs caused pumping rates in *C. orientalis* to decrease by up to 90%. This decrease was generally followed by a coordinated closure of the oscula and the cessation of pumping. However, longer term studies are still needed to confirm the effects of chronic sediment stress.

##  Supplemental Information

10.7717/peerj.2761/supp-1 Data S1 Fig. 3 raw data

10.7717/peerj.2761/supp-2 Data S2 Fig. 4 Raw data

10.7717/peerj.2761/supp-3 Data S3 Fig. 5 raw data

10.7717/peerj.2761/supp-4 Data S4 Figs. 6 and 7 raw data

## Additional Information and Declarations

### Field Study Permissions

The following information was supplied relating to field study approvals (i.e., approving body and any reference numbers): All collections were performed under GBR Marine Park Authority Permit:G13/35758.1.

### Data Availability

The following information was supplied regarding data availability: The raw data has been supplied as a Supplemental File.
